# Supplementary material for: A Critical View of the Application of the APEX Software (Aqueous Photochemistry of Environmentally-Occurring Xenobiotics) to Predict Photoreaction Kinetics in Surface Freshwaters
Source: Molecules. 2019 Dec 18;25(1):9. doi: 10.3390/molecules25010009 (PMC7017383; doi:10.3390/molecules25010009)
Supplement: Supplementary file 1 [file molecules-25-00009-s001.zip › 3.2.4_gcc-4.4.0.zip!/3.2.4_gcc-4.4.0/3.2.4_gcc-4.4.0/doc/octave/html/interpreter/Convex-Hull.html]

Convex Hull - Untitled


Next: Interpolation on Scattered Data,
Previous: Voronoi Diagrams,
Up: Geometry


---

### 29.3 Convex Hull

The convex hull of a set of points is the minimum convex envelope
containing all of the points. Octave has the functions `convhull`
and `convhulln` to calculate the convex hull of 2-dimensional and
N-dimensional sets of points.

— Function File: H = **convhull** (x, y)  
— Function File: H = **convhull** (x, y, opt)  
> Returns the index vector to the points of the enclosing convex hull. The
> data points are defined by the x and y vectors.
>
> A third optional argument, which must be a string, contains extra options
> passed to the underlying qhull command. See the documentation for the
> Qhull library for details.
>
>
>
> **See also:** delaunay, convhulln.

— Loadable Function: h = **convhulln** (p)  
— Loadable Function: h = **convhulln** (p, opt)  
— Loadable Function: [h, v] = **convhulln** (...)  
> Return an index vector to the points of the enclosing convex hull.
> The input matrix of size [n, dim] contains n points of dimension dim.
>
> If a second optional argument is given, it must be a string or cell array
> of strings containing options for the underlying qhull command. (See
> the Qhull documentation for the available options.) The default options
> are "s Qci Tcv".
> If the second output V is requested the volume of the convex hull is
> calculated.
>
>
>
> **See also:** convhull, delaunayn.

An example of the use of `convhull` is

```
     x = -3:0.05:3;
     y = abs (sin (x));
     k = convhull (x, y);
     plot (x(k), y(k), "r-", x, y, "b+");
     axis ([-3.05, 3.05, -0.05, 1.05]);
```
